# Prevalence of low back pain and its associated factors among traditional cloth weavers in Gulele sub-city, Addis Ababa, Ethiopia

**DOI:** 10.3389/fpubh.2023.1181591

**Published:** 2023-07-13

**Authors:** Amare Terfe, Temima Jemal, Tolossa Waqkene

**Affiliations:** ^1^Department of Environmental Health Science, College of Medicine and Health Sciences, Arba Minch University, Arba Minch, Ethiopia; ^2^Department of Environmental Health Science and Technology, Institute of Health, Jimma University, Jimma, Ethiopia; ^3^Department of Public Health, Dawo District Health Office, Woliso, Ethiopia

**Keywords:** low back pain, weavers, prevalence, musculoskeletal—disorders, factors

## Abstract

The prevalence of work-related musculoskeletal disorders particularly low back pain is significantly high among common informal or small-scale enterprises within developing countries like weaving. However, there is little information on risk factors for low back pain in the informal sector such as the weaving sector in the areas of Addis Ababa as well as in Ethiopia. Therefore this study was aimed to assess the magnitude of low back pain and its associated factors among traditional cloth weavers in the Gulele sub-city, Addis Ababa, Ethiopia. A cross-sectional study with internal comparison was conducted from June 1, 2022, and August 30, 2022. A total of 660 traditional cloth weavers were included in the study by using a systematic random sampling method to select participants in each of cooperatives as well as individual households in 5 woredas in Gulele sub-city. The data was analyzed and managed using SPSS version 20. A multivariate logistic regression analysis was used to control potential confounding factors and to determine the association. Among 660 (100%) respondents, 330 (50%) of them experienced low back pain after starting weaving. Out of the respondents with low back pain through their job career, 291 (44.1%) of them experienced low back pain during the last 12 months. Working greater than 8 h per day (AOR = 4.60, 95%CI: 2.35, 8.87), working with frequent bending (AOR = 3.32, 95%CI: 1.49, 7.40), job stress (AOR = 1.68, 95%CI: 1.18, 2.40) were among factors significantly associated with the occurrence of low back pain. This study has shown a high prevalence of low back pain among traditional cloth weavers which indicates the need for immediate public health action. However, very small improvements in the working condition, weaving tools design, working methods can potentially lead to large benefits.

## Introduction

Musculoskeletal disorders are injuries and disorders of the soft tissues and nervous system. They can affect almost any tissue, but the arms and back are the most commonly affected. Musculoskeletal disorders are one of the most common public health issues in today’s world, and they are caused by a variety of risk factors (1). It is common in many professions and the second leading cause of transient work injury after the common cold (2), as well as a source of workplace accidents and impairments in developing countries (3). Work-related musculoskeletal disorders (WMSDs) have long been recognized as a leading cause of nonfatal accidents and work-related absences in manufacturing communities (4).

Lower back pain (LBP) is the most common musculoskeletal disorder (MSD) affecting adults, with a lifetime prevalence of up to 84% (5). Symptoms can arise from a variety of anatomical sources, including nerve roots, muscles, fasciae, bones, joints, intervertebral discs (IVDs), and organs within the abdominal cavity (6). Previous research has found that LBP is responsible for an estimated 21.8 million disability adjusted life years (DALYs) in 2010. LBP affects approximately 60–80% of people at some point in their lives (7). In 2015, low back and neck pain were ranked as the fourth leading cause of DALYs worldwide. DALYs for low back and neck pain increased by 59.5% between 1990 and 2015. Low back and neck pain were the leading causes of disability in the majority of countries in 2015 (the second leading cause of DALYs in high-income countries after ischemic heart disease) (8).

Furthermore, lower back pain (LBP) is a leading cause of disability globally (1). It is one of the most common chronic disorders and has a significant economic impact worldwide (9). The pathophysiological causes of LBP are frequently unknown (10). This may lead to skepticism or dismissal of the seriousness and legitimacy of the disability associated with LBP (11). In addition to the physical effects that LBP patients experience, the condition has personal, societal, and psychological ramifications (12). Furthermore, chronic LBP is frequently accompanied by increased work absenteeism, lower productivity, status loss, and depressive symptoms (13).

A variety of factors influence the occurrence of chronic low back pain in various professions. Working postures are influenced by the shape, arrangement, size, and placement of the tools used as well as their operating methods. Unnatural body positions and non-ergonomic ways of working for an extended period of time can result in a variety of health issues for employees. Working in the same position for an extended period of time whether standing or sitting will cause discomfort (14). Working postures such as sitting for an extended period of time without any adjustment can soften the abdominal muscles, causing spine curvature and causing respiratory and digestive organ disorders (15).

Furthermore, a poor ergonomic workstation has been shown to increase muscular load and activity, resulting in an increase in MSD (16). Physical factors or loads on the biomechanical system are thought to cause tissue damage and inflammation, leading to MSD (17). Quantifying a workstation’s ergonomic risk is a method to cost-effectively implement and manage MSD in the workplace (18). However, there is still conflicting evidence that there is no link between workstation configuration and MSD (1, 19).

In developing countries such as Iran, China, Turkey, India, Pakistan, Russia, Egypt, Nepal, and Afghanistan, weaving is a common industry (20). The back is a vulnerable area in humans due to the mechanisms of the human body and the various types of tissue and structures that comprise the spine (21). Weavers weave in a variety of sitting postures, including forward flexion, upright posture, and side bending (22). During weaving, weavers sit continuously on a hard floor or a hard wooden bench with no back support. It stresses their lower limbs, calf muscles, and back. Throughout the weaving process, throwing shuttles and moving reed frames necessitate repetitive movements with no breaks. This could have long-term health implications. Weavers lean forward to avoid the effort required for rolling operations and maintain this posture for as long as weaving is possible, resulting in severe back pain. Furthermore, as raw material prices rise, the survival of the industry becomes increasingly difficult; as a result, weavers work long hours on the loom to complete tasks in order to earn more money (23, 24).

For centuries, Ethiopians have practiced traditional cotton weaving using both endogenous and exogenous technology. Cotton weavers work in small, cramped spaces under appalling working conditions. People working in the weaving industry are extremely vulnerable due to a lack of occupational safety and health services and poor working conditions because they are not supported by occupational safety and health services (25). Even in modern times, weaving has cultural value, and weavers must be properly cared for and valued as artisans. This research is critical not only for the health of weavers, but also for the esthetic and cultural value of the weaving profession. Traditional cloth weaving is still practiced in many parts of Ethiopia. The occupation has contributed significantly to the economies of both the workers in the sector and the country as a whole. There is, however, a scarcity of information about the health issues that weavers face at work. As a result, the purpose of this study was to determine the extent of low back pain and its associated factors among traditional cloth weavers in the Gulele sub-city of Addis Ababa, Ethiopia.

## Materials and methods

### Study setting and design

A descriptive cross-sectional study with internal comparison was conducted between June 1, 2022, and August 30, 2022, G.C. Weaving has long been a significant rural and urban home industry activity in Ethiopia. According to a handlooms cluster report, there are over 330,341 weavers’ nationwide and 66,068 weavers in Addis Abeba, with more than 60% (39,640) of them located in the Gulele sub-city of the Addis Ababa Administration. Weavers can be found at home, in sheds, and in cooperatives. Shiromeda, a sub-district on Addis Ababa’s northern outskirts near the Entoto Hills, is home to some of Ethiopia’s most celebrated weavers. Over the last 60 years, several weavers have migrated from southern Ethiopia to Shiromeda. Shiromeda and Addisu Gebeya are the traditional weaving hotspots in Addis Ababa (26). The study participants were drawn from Gulele, one of Addis Ababa’s 10 sub-cities.

### Sample size and sampling procedures

This study included all traditional cloth weavers from Addis Ababa’s Gulele sub-city who work in both individual households and cooperatives. The study subjects were weavers with more than 1 year of experience to determine the 12-month prevalence of low back pain among weavers. Because no similar studies in this sector had been conducted in Ethiopia or any other African country, the sample size was calculated using a single proportion formula with a prevalence of 50% since we could not get study conducted in Ethiopia and other similar countries in Africa on this topic. Furthermore, we calculated sample size for factors that contribute for the occurrence of low back pain among different professions in Ethiopia. Finally we used the larger sample size for the study since it is recommended to use the larger sample size to increase the representativeness of this study. The total sample size for this study was 660 weavers who worked in both individual households and government-established cooperatives.

**Figure 1:**
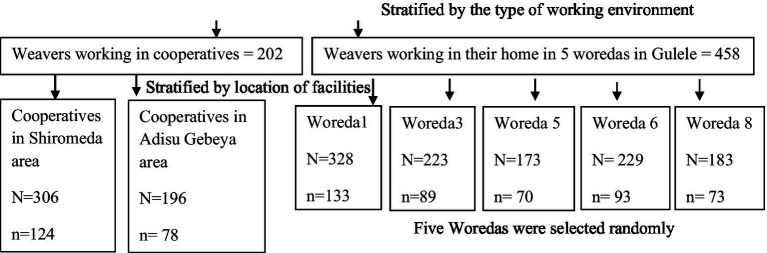
Sampling chart of weaving facilities.

The total sample size required for this study was proportionally allocated to each of the woredas and cooperatives found in Gulele sub city according to their weight (Figure 1).

The total sample size was stratified into individual households and cooperatives. A systematic random sampling method was used to select participants in Adisu Gebeya from 306 weavers organized into 11 cooperatives; 196 weavers organized into 7 cooperatives in the Shiromeda area; and individual households in 5 woredas in Gulele sub-city. The total sample size required for this study was proportionally allocated to each of the woredas and cooperatives found in the Gulele sub-city based on their weight.

### Data collection procedures

A structured questionnaire was used to collect data from traditional cloth weavers who work in both individual households and cooperatives. The questionnaire was designed using the Nordic standard musculoskeletal questionnaire and was structured to include both the responses of the respondents and the observations of the interviewers. The data collectors and supervisor were given 2 days of training on how to conduct interviews, study subjects’ rights, and ethical issues such as confidentiality. Following data collector training, the questionnaire was pretested with 5% of the sample in areas that were unable to be included in the actual study, and the data collection process was supervised by two supervisors to ensure data quality and validity.

### Data quality management and analysis

The collected data was checked for errors before being transferred to SPSS 26 software for analysis and data management. The data were analyzed using the SPSS statistical package for percentage calculations, and Univariate analysis was used to describe the data using percentages, and bivariate and multivariate analysis to determine the presence of association. In the bivariate analysis, variables with a 0.25 probability of association with the outcome variable were entered into a multivariate logistic regression to control for potential confounding factors. An odds ratio with a 95% confidence interval and a *p*-value of 0.05 was used to determine the presence of a significant association.

### Ethical consideration

The University of Gondar’s ethical review board provided approval and clearance, as did the Gulele sub-city Small Scale Enterprise Development Agency. Furthermore, each study participant provided verbal informed consent. Respondents were informed that they could skip or end the interview process at any time. Furthermore, the study participants were informed that any information obtained from them would be kept confidential and used solely for research purposes.

## Result

### Socio-demographic characteristics of traditional cloth weavers

A total of 660 (100%) respondents took part in this study, with a response rate of 100%. Among the study participants, 591 (89.5%) were males, while only 69 (10.5%) were females. The majority of respondents, 256 (38.8%), were between the ages of 30 and 41, with a mean (±SD) age of 35.2(±11.921) years. The majority of study participants [341 (51.7%) had a high school (7–10) education, while the remaining 69 (10.5%) were uneducated (Table 1)].

**Table 1:** Socio-demographic characteristics of traditional cloth weavers in the Gulele sub-city, Addis Ababa, Ethiopia, 2022.

Variables		Frequency	Percent (%)
Sex	Male	591	89.5
	Female	69	10.5
Age	<30	249	37.7
	30–41	256	38.8
	42–53	91	13.8
	54–65	47	7.1
	>65	17	2.6
Educational status	Illiterate	69	10.5
	Primary school (1–6)	156	23.6
	High school (7–10)	341	51.7
	Preparatory (11–12)	31	4.7
	University/college	63	9.5
Marital status	Married	199	30.2
	Single	457	69.2
	Divorced	4	0.6
Monthly income	≤750 birr	76	11.5
	>750 birr	584	88.5
Work experience	≤10 years	267	40.5
	˃10 years	393	59.5

### Prevalence of low back pain among traditional cloth weavers

Among 660 respondents, 330 (50%) reported low back pain after beginning to weave (95%CI: 46.2, 53.9%). About 291 (44.1%) of respondents with low back pain during their job career experienced low back pain in the previous 12 months (95%CI: 40.3, 47.7%). Among weavers who reported having low back pain in the previous 12 months, 68 (10.3%) of them felt low back pain during the last 7 days. In terms of work absenteeism, 212 (32.1%) of the respondents were absent due to low back pain, with 102 (48.1%), 59 (27.8%) of the weavers were absent for 1–7 and more than 30 working days consecutively during the previous 12 months (Table 2).

**Table 2:** Prevalence of low back pain among traditional cloth weavers in the Gulele sub-city, Addis Ababa, Ethiopia, 2022.

Variables		Number	Percent
History of low back pain	Yes	4	0.6
	No	656	99.4
Low back pain after starting weaving	Yes	330	50
	No	330	50
Low back pain in the last 12 months	Yes	291	44.1
	No	369	55.9
Duration of the low back pain	1 Day	35	5.3
	2–7 Days	107	16.2
	More than a week	115	17.4
	Do not recall	34	5.2
Low back pain in the last 7 days	Yes	68	10.3
	No	592	89.7
Hospitalization due to low back pain	Yes	149	22.6
	No	511	77.4
Frequency of Hospitalization due to low back pain	Once	23	15.4
	More than once	126	84.6
Changing jobs due to low back pain	Yes	34	5.2
	No	626	94.8
Reducing activity due to low back pain	Yes	215	32.6
	No	445	67.4
Duration of reducing activity due to low back pain	0 day	453	68.6
	1–7 days	96	14.5
	8–30 days	55	8.3
	More than 30 days	56	8.5
Absent from work due to low back pain	Yes	212	32.1
	No	448	67.9
Duration absent from work due to low back pain	1–7 days	102	48.1
	8–30 days	51	24.1
	More than 30 days	59	27.8
Feeling low back pain during sitting	Yes	270	40.9
	No	390	59.1
Feeling low back pain during standing	Yes	91	13.8
	No	569	86.2

### Socio-demographic determinants

Bivariate and multivariate analyses of socio-demographic determinants were performed using the logistic regression model. The bivariate logistic regression analysis revealed a statistically significant association between age group, educational status, monthly income, work experience, and low back pain. In the multivariate logistic regression analysis, low back pain was significantly associated only with lower monthly income (AOR 1.73, 95%CI 1.01, 2.95) and illiteracy (AOR 3.10, 95%CI 1.33, 7.20; Table 3).

**Table 3:** Association between socio-demographic determinants and low back pain among traditional cloth weavers in the Gulele sub-city, Addis Ababa, Ethiopia, 2022.

Variables		Low back pain		COR (95% CI)	AOR (95% CI)	*p*-value
		Yes	No			
Sex^©^	Male	256	335	1		
	Female	35	34	1.35 (0.82,2.22)		
Age	<30	91	158	1	1	
	30–41	107	149	1.25 (0.87, 1.78)	1.08 (0.71, 1.62)	0.726
	42–53	50	41	2.12 (1.30, 3.45)**	1.46 (0.81, 2.64)	0.205
	54–65	29	18	2.80 (1.47, 5.32)**	1.59 (0.73, 3.46)	0.244
	>65	14	3	8.10 (2.27, 28.95)***	2.93 (0.72, 11.96)	0.135
Educational status	Illiterate	51	18	5.28 (2.50, 11.14)***	3.10 (1.33, 7.20)**	**0.009**
	Primary school (1–6)	66	90	1.37 (0.74, 2.51)	0.97 (0.50, 1.86)	0.919
	High school (7–10)	138	203	1.27 (0.72, 2.22)	1.16 (0.65, 2.07)	0.626
	Preparatory (11–12)	14	17	1.54 (0.64, 3.69)	1.25 (0.49, 3.19)	0.640
	University/college	22	41	1	1	
Monthly income	≤750 birr	45	31	1.99 (1.23, 3.24)**	1.73 (1.01, 2.95)*	**0.044**
	>750 birr	246	338	1	1	
Work experience	≤10 years	98	169	1	1	
	˃10 years	193	200	1.66 (1.21, 2.29)**	1.21 (0.81, 1.80)	0.348

Significant at *p ≤ 0.05, **p ≤ 0.01, ***p ≤ 0.001.

^©^Not included in the multivariate analysis.

The bold values showed the independent variables are significantly associated with the outcome variable.

### Working condition and environmental determinants

Among working condition and environmental determinants, working for more than 8 h per day (AOR = 4.60, 95%CI 2.35, 8.87), working with frequent bending (AOR = 3.32, 95%CI 1.49, 7.40), working with uncomfortable posture (AOR =2.07, 95%CI 1.23, 3.49) and tasks creating pressure on the back (AOR = 4.41, 95%CI 1.87, 10.41) remained significant in the multivariate analysis (Table 4).

**Table 4:** Association between working condition and environment determinants and low back pain among traditional cloth weavers in the Gulele sub-city, Addis Ababa, Ethiopia, 2022.

Variables		LBP		COR (95% CI)	AOR (95% CI)	*p* value
		Yes	No			
Working hours per day	≤8 h	13	66	1	1	
	˃8 h	278	303	4.66 (2.51,8.63)***	4.60 (2.35,8.87)***	**0.001**
Workplace comfort	Yes	141	271	1	**1**	
	No	150	98	2.94 (2.12,4.08)***	1.32 (0.89,1.96)	0.164
Presence of chair with back support	Yes	14	91	1	1	
	No	277	278	6.48 (3.60,11.65)***	1.98 (0.78,4.97)	0.149
Presence of comfortable seat	Yes	10	88	1	1	
	No	281	281	8.80 (4.48,17.28)***	2.17 (0.78,5.99)	0.136
Work with twisted back for a long time	Yes	172	176	1.59 (1.16,2.16)***	1.06 (0.64,1.77)	0.817
	No	119	193	1	1	
Work with frequent bending	Yes	279	297	5.64 (2.99,10.61)***	3.32 (1.49,7.40)**	**0.003**
	No	12	72	1	1	
Sitting for a prolonged time	Yes	288	353	4.35 (1.26,15.08)**	0.60 (0.13,2.66)	0.498
	No	3	16	1	1	
Working with uncomfortable posture	Yes	205	212	1.77 (1.28,2.45)***	2.07 (1.23,3.49)**	**0.006**
	No	86	157	1	1	
Working in the same static posture	Yes	281	327	3.61 (1.78,7.33)***	3.57 (0.35,36.85)	0.286
	No	10	42	1	1	
Adjusted work station	Yes	13	102	1	1	
	No	278	267	8.17 (4.48,14.91)***	1.92 (0.87,4.21)	0.104
Task creating pressure on the back of weavers	Yes	283	272	12.62 (6.02,26.44)***	4.41 (1.87,10.41)***	**0.001**
	No	8	97	1	1	

Significant at *p ≤ 0.05, **p ≤ 0.01, ***p ≤ 0.001.

The bold values showed the independent variables are significantly associated with the outcome variable.

### Behavioral and psychosocial determinants

Among behavioral and psychosocial determinants, taking a break and resting time during the workday did not show a significant association with the occurrence of low back pain in the bivariate analysis. However, practicing regular physical activities (AOR = 1.77, 95%CI 1.21, 2.57), knowledge about the causes of low back pain (AOR = 4.49, 95%CI 3.14, 6.41), job stress (AOR = 1.68, 95%CI 1.18, 2.40), and job dissatisfaction (AOR = 1.63, 95%CI 1.13, 2.36) were significantly associated with the occurrence of low back pain (Table 5).

**Table 5:** Association between behavioral and psychosocial determinants and low back pain among traditional cloth weavers in the Gulele sub-city, Addis Ababa, Ethiopia, 2022.

Variables		Low back pain		COR (95% CI)	AOR (95% CI)	*p* value
		Yes	No			
Taking a break	Yes	116	223	1	1	
	No	175	146	2.30 (1.68, 3.15)***	0.89 (0.05, 14.73)	0.936
Resting time in a working day	No break	175	146	3.30 (1.03, 10.57)*	3.22 (0.15, 68.58)	0.453
	1 h	98	180	1.40 (0.46, 4.83)	1.32 (0.39, 4.49)	0.662
	2 h	14	32	1.20 (0.33, 4.44)	1.38 (0.33, 5.26)	0.696
	>3 h	4	11	1	1	
Regular physical activities	Yes	87	151	1	1	
	No	204	218	1.62 (1.17, 2.25)***	1.77 (1.21, 2.57)**	**0.003**
Knowledge about the cause of low back pain	Yes	118	265	1	1	
	No	173	104	3.74 (2.70, 5.18)***	4.49 (3.14, 6.41)***	**0.001**
Job stress	Yes	154	132	2.02 (1.48, 2.76)***	1.68 (1.18, 2.40)**	**0.004**
	No	137	237	1	1	
Job satisfaction	Yes	78	155	1		
	No	213	214	1.98 (1.42, 2.76)***	1.63 (1.13, 2.36)**	**0.010**

Significant at *p ≤ 0.05, **p ≤ 0.01, ***p ≤ 0.001.

The bold values showed the independent variables are significantly associated with the outcome variable.

## Discussion

Weavers in small-scale industries and informal sectors had significantly higher rates of low back pain (27). Furthermore, traditional cloth weaving is one of the informal sectors that contribute significantly to the Ethiopian economy and its workers. However, the health issues associated with this sector did not receive the attention they deserved, and no research was conducted. As a result, the goal of this study was to determine the prevalence of low back pain and its associated factors among traditional cloth weavers in Addis Ababa’s Gulele sub-city.

According to the study’s findings, the prevalence of low back pain among traditional cloth weavers was 330 (50%) after they began weaving (95% CI: 46.2, 53.9%). The 12-month prevalence among study participants was 291 (44.1%) (95% CI: 40.3, 47.7%), which is comparable to studies done among Ethiopian young traditional cloth weavers (48.9%) and women weavers working with handlooms in Samarinda, Indonesia (28, 29). However, the prevalence of LBP is lower than in studies conducted in the weaving industries of Uttarakhand, Arunachal Pradesh, and Varanasi in India and Northern Thailand, where the prevalence of LBP was 67.19, 79.2, 82.91, and 81%, respectively (22, 24, 30, 31). The difference in the prevalence of LBP in these two studies could be attributed to differences in the working conditions of weavers. The Indian study focused on well-organized workshops with a poor working environment, whereas this study focused on individual households and cooperatives with less organized workshops. Furthermore, working conditions differ between handloom and power loom weaving in India and traditional cloth weaving in Ethiopia.

**Figure 2:**
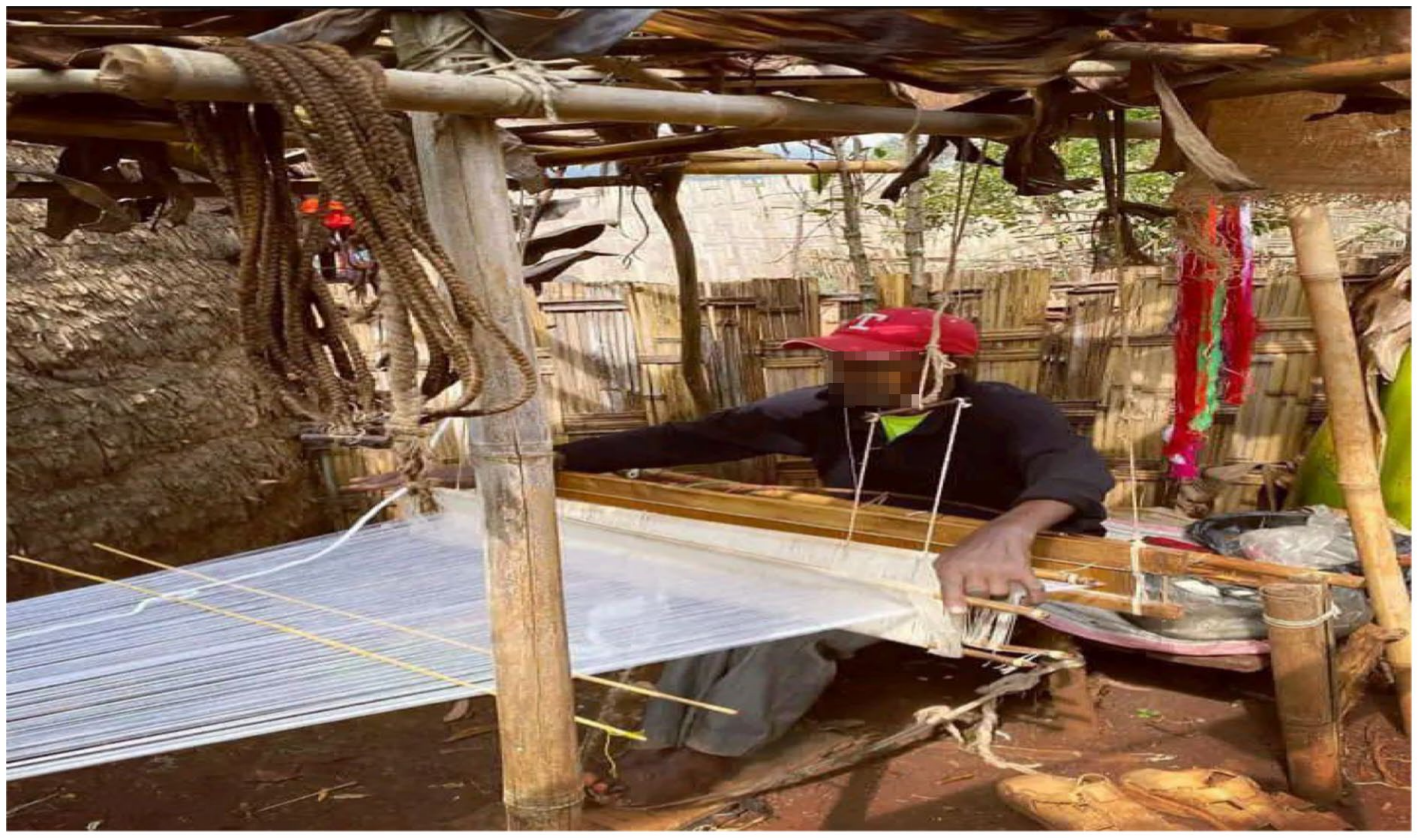
Sampling chart of weaving facilities.

Weavers suffer from musculoskeletal disorders for a variety of reasons, the most important of which is the prolonged use of a constrained sitting posture (Figure 2). LBP was the most common complaint among those suffering from musculoskeletal disorders. According to research, weavers frequently experience pain in their lower back (32–35). Multiple factors may contribute to the occurrence of low back pain among weavers. According to this study, one of the factors that may contribute to the occurrence of LBP is illiteracy. According to this study, illiterate weavers were 3.10 times more likely than those with a college education or higher to develop LBP (AOR = 3.10, 95% CI: 1.33, 7.20). This finding was consistent with previous research conducted among Iranian petrochemical industry workers and women weavers using handlooms in Samarinda, Indonesia (28, 36). This study, on the other hand, contradicted a study conducted among textile factory workers in Amhara regional state, which discovered that educational status was not significantly associated with LBP (37).

The number of working hours per day was another factor that could contribute to the occurrence of LBP among traditional cloth weavers. Weavers working more than 8 h per day were 4.60 times more likely to develop LBP than those working less than or equal to 8 h per day (AOR = 4.60, 95% CI: 2.35, 8.87). Furthermore, this study was consistent with previous research conducted among weavers in Varanasi, India, LetmafoInduk Village, Insana Tengah District, Indonesia, and women weavers working with handlooms in Samarinda, Indonesia, which discovered that daily working time had a positive association with LBP among weavers (28, 30, 34). The reason for this could be that daily working hours in the weaving industry are not usually fixed; they vary depending on the situation and workload, and the desire to earn more money drives weavers to work longer hours. Furthermore, the long working hours cause fatigue of the physical endurance of the muscles and bones, resulting in low back pain (32). This study, however, contradicted previous research conducted among textile industry workers in Pakistan, weavers in Varanasi, India, and weavers in Pathrail union in Tangail district, Bangladesh, which discovered that working hours were not significantly associated with the occurrence of low back pain (30, 38, 39).

**Figure 3:**
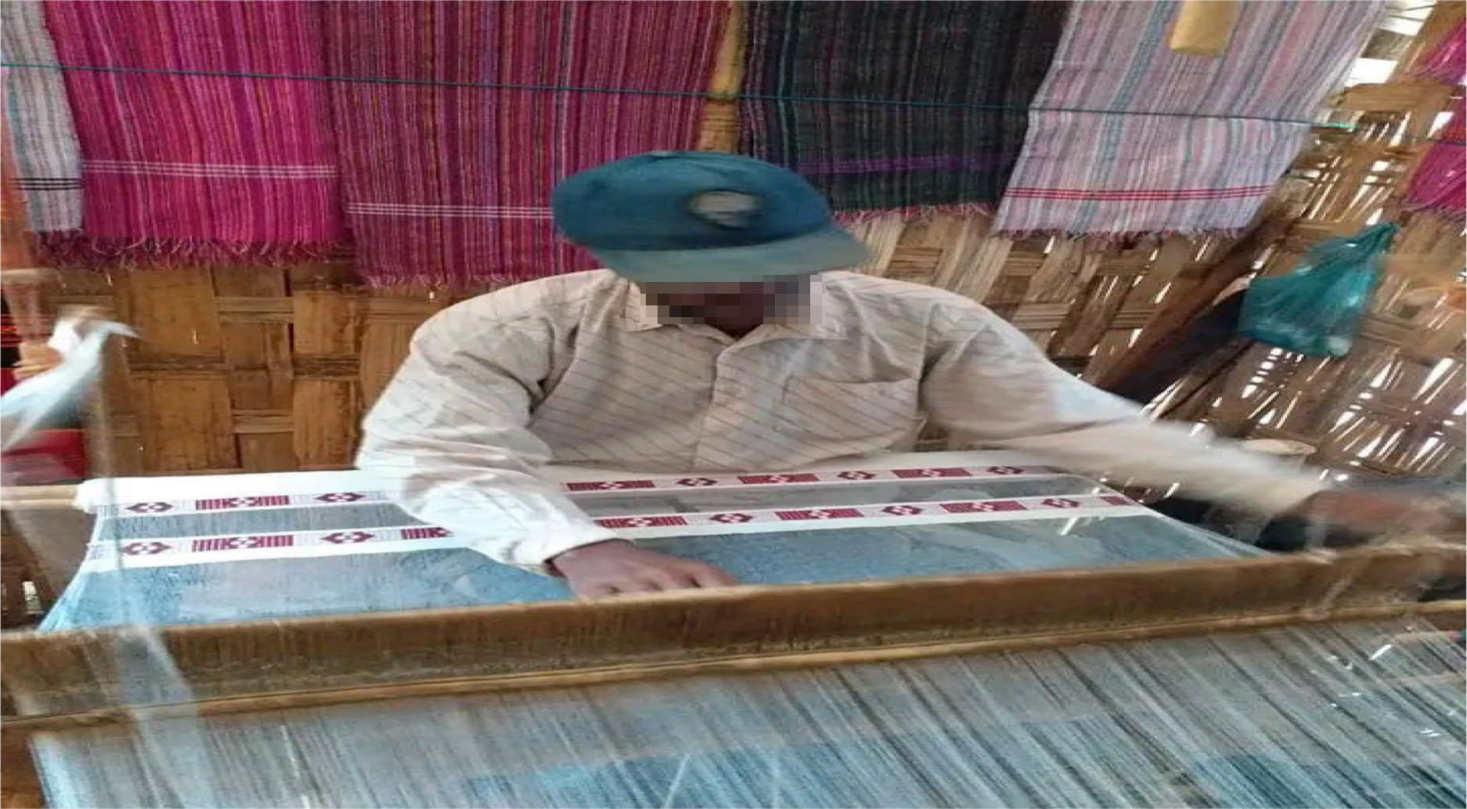
Working with the trunk bent for longer duration.

Several occupational risk factors are associated with the weaving method, including the requirement to sit for long periods of time with the trunk forward bent (Figure 3), as well as activities such as pulling, pushing, lifting tools, working while bent or twisted at the waist, and repetitive motions with hands/wrists and gripping (22, 24). Similarly, weavers who worked with frequent bending were found to be 3.32 times more likely to report LBP than those who did not (AOR = 3.32, 95%CI: 1.49, 7.40). This is because frequent bending involves the same joints and muscle groups, and when workers perform the same motion too frequently, too quickly, and for too long, frequent bending becomes dangerous to the weavers’ backs (22, 23, 40).

**Figure 4:**
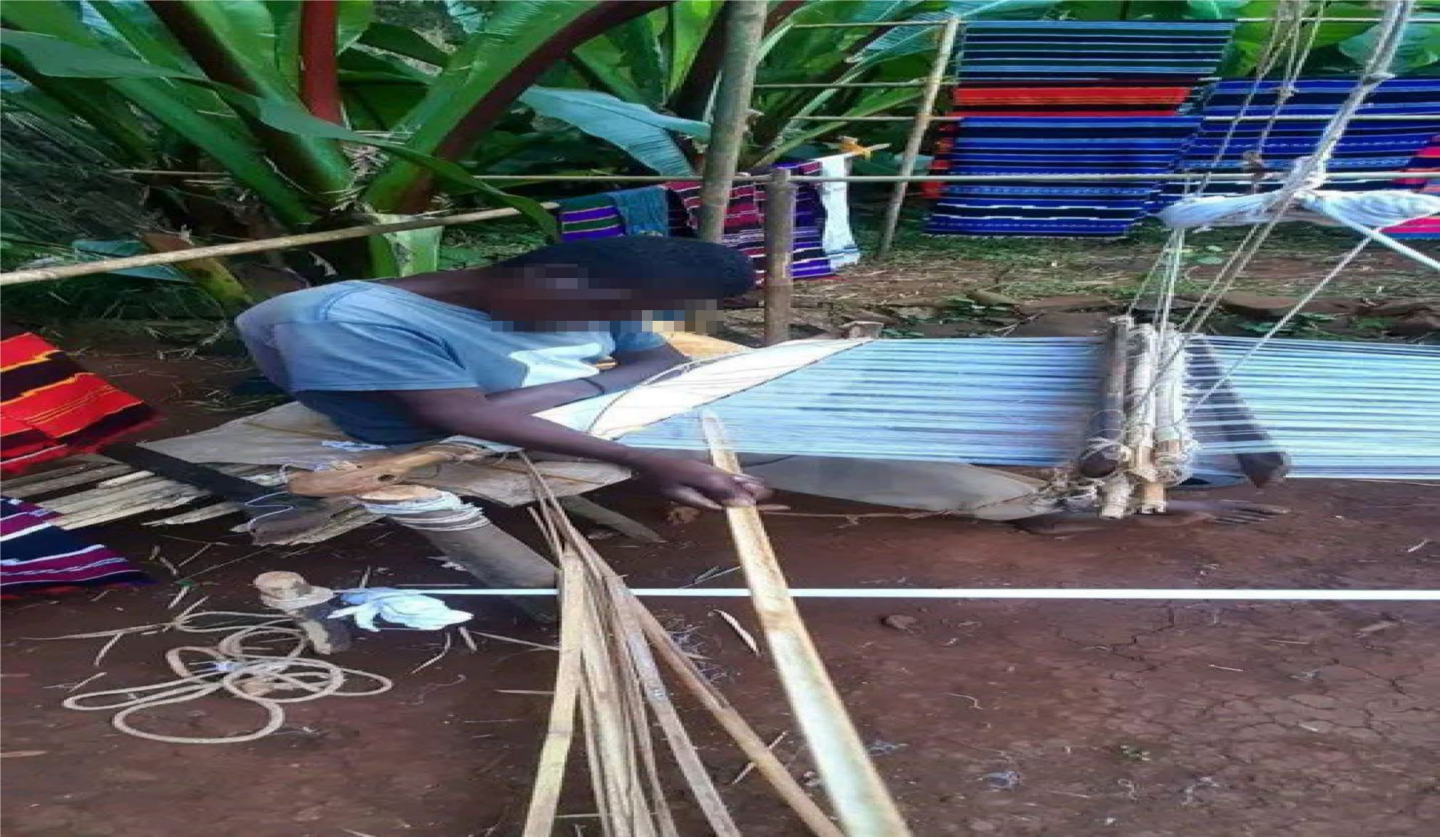
Working using a variety of posture including forward flexion and side bending.

Weavers typically work while seated (Figure 4). Weavers’ use a variety of sitting postures while weaving, including forward flexion, upright posture, and side bending (22). Several studies in various professions, including weaving, have revealed that working in an awkward posture contributes to the occurrence of musculoskeletal problems in various body regions (21, 41). Similarly, weavers who worked in an uncomfortable posture were about 2.07 times more likely than those who did not work in an uncomfortable posture to report LBP (AOR = 2.07, 95% CI: 1.23, 3.49). Given that weavers used to weave for long periods of time while seated, with a stretched, extended body and repeatable movement, the outcome is not surprising. Furthermore, several studies among weavers discovered that bending, twisting, and static postures that put pressure on the back were risk factors for LBP (22, 24, 42). Weaving looms, for example, have a sitting arrangement with no cushion or back support, which weavers have reported as uncomfortable. It strains the lower limbs, calf muscles, and back (40, 42).

The perception of weaving as stressful, physically and mentally demanding, a lack of adequate breaks, and the presence of long working hours have all contributed to weavers’ stress levels. Weavers who were stressed were 1.68 times more likely to complain of LBP than their counterparts (AOR = 1.68, 95% CI: 1.18, 2.40). A study of textile factory workers in Ethiopia’s Amhara regional state, as well as a study of young weavers in Ethiopia, who reported working under pressure and emotional abuse and were nearly twice as likely to develop LBP, backed up this finding (29, 37).

Finally, satisfaction with the working environment, current job, income from current job, and community status all played a role in the occurrence of LBP. Weavers who were dissatisfied were 1.63 times more likely than their counterparts to experience LBP (AOR = 1.63, 95% CI: 1.13, 2.36). A study conducted among weavers in Varanasi, supports this finding (30).

## Conclusion

This study discovered a high prevalence of low back pain among traditional cloth weavers, indicating the need for immediate public health action to improve Ethiopian traditional cloth weavers’ health. Having a higher income, taking breaks, engaging in regular physical activity, and having a job that you enjoy were all factors that could reduce your risk of LBP. Furthermore, working for more than 8 h per day, frequent bending, working in an uncomfortable posture, tasks that put pressure on the weaver’s back, and job stress were all linked to an increased risk of LBP. As a result, ergonomically oriented weaving workstations are required because the majority of WRMSDs are caused by poorly designed workstations. Even minor changes in working conditions, weaving tool design, and working methods can result in significant benefits, such as reduced bending, uncomfortable posture, and tasks that put strain on weavers’ backs. As a result, concerned stakeholders must act to improve working conditions in the traditional weaving sector in order to improve both weavers’ health and productivity of the weaving sector.

## Limitation of the study

The Nordic Musculoskeletal Disorder Questionnaire cannot determine the risk level of low back pain symptoms, which is a limitation of this study. This study did not analyze the risk of low back pain symptoms among traditional cloth weavers because it was not the study’s goal. As a result, other researchers can investigate Musculoskeletal Disorder Symptom Risk Levels using methods other than the NMQ.

## Data availability statement

The raw data supporting the conclusions of this article will be made available by the authors, without undue reservation.

## Ethics statement

The studies involving human participants were reviewed and approved by University of Gondar review committee. The patients/participants provided their written informed consent to participate in this study.

## Author contributions

AT has been contributed to idea inception, design methodology, data entry, data analysis, and manuscript preparation for publications. TJ and TW has been contributed to data collection, data entry, data analysis, and manuscript preparation for publications. All authors contributed to the article and approved the submitted version.

## Conflict of interest

The authors declare that the research was conducted in the absence of any commercial or financial relationships that could be construed as a potential conflict of interest.

## Publisher’s note

All claims expressed in this article are solely those of the authors and do not necessarily represent those of their affiliated organizations, or those of the publisher, the editors and the reviewers. Any product that may be evaluated in this article, or claim that may be made by its manufacturer, is not guaranteed or endorsed by the publisher.
